# Community-associated Carbapenem-Resistant Organism Case Investigations in New York City, December 2020-May 2023

**DOI:** 10.1017/ash.2024.323

**Published:** 2024-09-16

**Authors:** Celina Santiago, Ying Lin, Ulrike Siemetzki-Kapoor, Nicole Burton, Katelynn Devinney, Balan Dominique, Portier Thomas, William Greendyke, Molly Kratz, Catharine Prussing, Kailee Cummings, Rebecca Zimba, Karen Alroy

**Affiliations:** NYC Department of Health and Mental Hygiene; Wadsworth Center, NYSDOH; CUNY Institute for Implementation Science in Population Health

## Abstract

Epidemiology of carbapenem-resistant organisms (CRO) has focused on transmission in acute care hospital or long-term care facility (LTCF) settings. Few investigations have examined community-associated (CA)-CRO, with no consensus about common exposures. To explore possible exposures, the New York City (NYC) Department of Health and Mental Hygiene investigated suspected CA-CRO cases through routine surveillance among NYC residents with specimens collected during December 2020-May 2023. CA-CRO cases were defined as urine or skin specimens with bacterial cultures exhibiting carbapenem resistance, among individuals aged ≤70 years with no international travel, hospitalization, or LTCF stays within 12 months before specimen collection. Inclusion was determined by reviewing data from health information exchanges, when available electronic medical records, and telephone screening for those not excluded through record review. We identified 426 suspected cases for review, those not meeting the case definition were excluded; 44 individuals were not reached for screening. A preliminary questionnaire was fielded with 12 individuals and then refined to capture additional potential exposures. Analyses were completed with 23 individuals interviewed with the refined questionnaire. Of the 23, 70% were female; 39% were Hispanic, 17% Black, and 17% White; their median age was 60 years (range: 26-70 years). Further, 83% reported an outpatient appointment, 48% reported an outpatient procedure/surgery, and 9% reported having a hospitalized household member, all within 12 months before specimen collection; 26% had a urinary catheter or indwelling device within 2 days of specimen collection. Additionally, 30% reported taking antibiotics within 3 months of specimen collection, 52% denied taking antibiotics, 9% were unsure about antibiotic use, and 9% did not answer the question. Whole genome sequencing (WGS) was performed on 14 available isolates from CA-CRO cases by the NYC Public Health Laboratory or Wadsworth Center (WC), of which only 7 could be compared with isolates previously sequenced at WC (2017-2023). Six isolates were separated by >50 mutation events, suggesting no close genomic relationship. One isolate from 2021 was 11 mutation events from a 2018 isolate from the same individual, consistent with the expected evolutionary rate. While infrequent, CA-CRO cases occur in NYC. Outpatient healthcare, antibiotic use, and urinary catheters or indwelling devices were common self-reported exposures. Analyses were limited by screening non-response. Increased specimen availability for WGS could enhance investigation of CA-CRO exposure patterns. Health information exchange data were often incomplete and future surveillance could benefit from healthcare and public health partnerships and better documentation for more complete electronic medical histories.

